# Clinical Characteristics of Hepatitis B Virus Patients After Switching to Tenofovir Alafenamide Fumarate: A Retrospective Observational Study

**DOI:** 10.7759/cureus.10380

**Published:** 2020-09-11

**Authors:** Abdullah S Alghamdi, Hammad S Alothmani, Mohammed Mogharbel, Hazeez Albiladi, Mohamed Babatin

**Affiliations:** 1 Medical Department/Gastroenterology Unit, King Fahad General Hospital, Jeddah, SAU; 2 Gastroenterology Unit, King Fahad Hospital, Jeddah, SAU

**Keywords:** treatment of hbv, tenofovir alafenamide fumarate, retrospective study

## Abstract

Background

Hepatitis B Virus (HBV) continues to be a significant global health problem despite vaccination programs and effective antiviral drugs.

Aim

Assess tenofovir alafenamide fumarate (TAF) as a new treatment modality in light of the clinical characteristics of HBV patients.

Settings and design

A real-world observational study

Methods and material

We collected data of 71 HBV patients and recorded the hepatitis B virus deoxyribonucleic acid (HBV-DNA) plasma levels and biochemistry test results for the alanine aminotransferase (ALT), aspartate aminotransferase (AST), and serum creatinine levels at three time points, including baseline, time of switching to TAF, and six months after switching.

Results

From the time of switching to TAF till six months later, HBV-DNA plasma levels significantly decreased from 838.61 IU/mL to 16.7 IU/mL (p-value of <0.05). ALT and AST levels dropped from 29.05 U/L to 27 U/L and from 21.34 U/L to 20.7 U/L (p-values 0.328 and 0.410, respectively). Although TAF did not show a statistically significant reduction in the serum levels of AST, ALT, and creatinine, it showed a detectable maintenance level.

Conclusions

In the evaluated cohort, all clinical characteristics of HBV were maintained six months after switching patients to TAF.

## Introduction

Hepatitis B virus (HBV) is a common blood-borne pathogen that continues to be a significant global health concern [1]. Despite effective vaccination programs, up to 250 million people around the world are still estimated to be positive for hepatitis B surface antigen (HBsAg) [2]. In the Eastern Mediterranean region, 3.3% of the adult population are living with HBV, according to the World Health Organization (WHO) [3]. Nonetheless, owing to the improvement in preventive and care measures, the Kingdom of Saudi Arabia (KSA) witnessed a dramatic decline in the prevalence of chronic HBV infection from 8.3% in 1988 to 1.3% in 2013 [4-5]. A chronic HBV infection is a major cause of morbidity and mortality, with almost 800,000 deaths annually. Without treatment, chronic hepatitis B can progress to cirrhosis, hepatocellular carcinoma (HCC), or liver failure [6]. Suppressive antiviral agents demonstrated a significant reduction in the risk of liver-related complications and may delay or reverse the progression of the disease [7-8].

Tenofovir (TFV) is a nucleotide analog that inhibits the reverse transcription of HBV (NRTI) [9]. Tenofovir alafenamide fumarate (TAF) is a new prodrug of TFV that was designed to have greater plasma stability at a lower dose than tenofovir disoproxil fumarate (TDF), allowing successful delivery of the tenofovir-diphosphate (TFV-DP) to hepatocytes [10]. High systemic exposure to TFV in the form of TDF was associated with bone and renal toxicities [11]. Therefore, replacing TDF with TAF could reduce the circulating level of TFV by up to 90% [12].

Recent recommendations by both the European Association for the Study of Liver (EASL) and the American Association for the Study of Liver Diseases (AASLD) released in 2017 and 2018 included TAF as one of the first‐line agents for HBV [13-14]. According to the recommendations released by the Saudi Association for the Study of Liver Diseases and Transplantation (SASLT) in 2014, the recommended anti-HBV agents in the country were entecavir (ETV) and TDF, along with lamivudine (LAM), telbivudine, adefovir, and pegylated interferon [15].

This observational study aimed to highlight the feasibility of TAF, after switching from other antiviral drugs including TDF, and its role in maintaining the clinical characteristics of HBV patients within the normal range.

## Materials and methods

Patients and methods

Study Design

This study was a retrospective, observational cohort study conducted on HBV-infected patients from a single center in Saudi Arabia. All data were collected from patient records available between December 2006 and November 2017. We included records of patients who met the following criteria: (1) age ≥ 18 years and < 80 years; (2) a confirmed diagnosis of chronic HBV infection; (3) patients who received oral antiviral (OAV) treatment for at least six months (± two weeks) prior to switching to TAF as per routine medical practice; and (4) patients attending the study site clinic with available patient records before and after switching to TAF. Patients were not eligible for this study if they fulfilled any of the following exclusion criteria: (1) incomplete data related to the variables of the primary endpoints; (2) creatinine clearance of ≤50 ml/min, estimated by the Cockcroft-Gault formula [16]; (3) history of organ transplantation; (4) co-infection with hepatitis C virus, hepatitis D virus, or human immunodeficiency virus (HIV); (5) HCC; or (6) alcohol abuse.

This study followed the Strengthening the Reporting of Observational Studies in Epidemiology (STROBE) statement guidelines for the reporting of observational studies [17]. The protocol was reviewed and approved by the hospital ethical committee prior to study initiation. Informed consent was not required due to the retrospective nature of the study and considering that there was no associated risk. The data were collected anonymously; therefore, no patient identifier is revealed. 

Data collection

Data were collected from the medical records of enrolled patients using an electronic case report form (CRF). Patient data were handled in compliance with all applicable privacy laws. Initial data collection was performed for each patient as per the schedule of events. Data were collected retrospectively at three time points: baseline (T1), at the time of the switch to TAF (T2), and at six (±0.5) months after switching to TAF (T3).

The study collected the following data at the three-time points: (1) demographic information: (age, gender, race); (2) relevant medical history, including the history of HBV diagnosis and management, and information regarding underlying diseases; (3) performed physical examination; any abnormal physical exam findings were documented; (4) body weight and body mass index (BMI); (5) vital signs (temperature, blood pressure, and pulse rate); (6) virology test results; HBV deoxyribonucleic acid (DNA) polymerase chain reaction (PCR) quantitative (HBV-DNA Quantitative by COBAS TaqMan (Roche Molecular Diagnostics Inc., Pleasanton, California) with a detection rate of less than 20 IU/ML), hepatitis B envelope antigen (HBeAg; BEPIII machine from Siemens, Munich, Germany); (7) FibroScan (ECHOSENS Company, Paris, France) results that were classified according to the stage of fibrosis (F0-F4) [18]; (8) biochemistry test results; alanine transaminase (ALT), aspartate transaminase (AST), serum creatinine, serum albumin, total bilirubin, phosphorous, calcium, magnesium, and international normalized ratio (INR); (9) complete blood count test results; (10) abdominal ultrasound findings; (11) associated co-morbidities; and (12) any concomitant medications to be collected, including name, dose, and frequency.

Statistical analysis

Descriptive statistics were used to present the baseline demographic data and clinical characteristics at the three time points of the study. Categorical variables were presented by counts and percentages, whereas continuous variables were presented by mean and standard deviation, in case of the normal distribution, or by the median and interquartile range (IQR) otherwise. Numerical data were explored for normality using the Kolmogorov-Smirnov test and the Shapiro-Wilk test. A paired t-test was used if the numeric data were normally distributed or non-parametric alternatives of the Wilcoxon signed-rank test if data were skewed. Categorical variables underwent a test of association using the McNemar test for paired data or Fisher’s exact test. The significance level was two-sided, with a type 1 error of 5%. The analyses were done using Statistical Package for Social Sciences version 24 (SPSS-24; IBM Corp., Armonk, New York).

## Results

In this single-center study, a total of 77 subjects met the eligibility criteria; however, only 71 subjects were enrolled in the analysis. Two patients were removed from the analysis as they did not complete six months on the last antiviral medication before switching to TAF, and four patients were excluded due to missing information.

Baseline characteristics of the study population

The mean age of the study population was 45± 12 years. Most of the patients were Arabs, and both genders were represented equally (Table 1). Patients enrolled in the study were confirmed to be diagnosed with chronic HBV infection, with a mean disease duration of 13.65±6.5 years. The mean HBV DNA levels at baseline were high (> 2x106 IU/mL) while most of the patients (88.7%) were negative for HBeAg. The mean levels of AST, ALT, and serum creatinine were within the normal range of each parameter (Table 1). Baseline FibroScan results were not available in 39 of the 71 enrolled patients. Regarding the fibrosis stage at baseline, the majority of patients had F1 stage fibrosis (18.3%), followed by F4 (12.7%), F0 (8.5%), F3 (8.5%), and F2 (7.0%). Liver ultrasound showed that more than half of the patients (53.5%) had normal liver ultrasonography while approximately 20% of patients had the ultrasonographic features of fatty liver and 15.5% of patients had the ultrasonographic signs of cirrhosis (Table 1)

**Table 1:** Baseline characteristics of the study population Categorical data were presented by frequency and percentage; Continuous data were presented as mean±SD HBV: hepatitis B virus; HBeAg: hepatitis B envelope antigen; ALT: alanine transaminase; AST: aspartate transaminase

Demographic variables	
Age, years (Mean± SD)	45± 12
Gender (n, %)	
Male	40 (56.3%)
Females	31 (43.7%)
Race (n, %)	
Arab	70 (98.6%)
non-Arab	1 (1.4%)
Medical History	
Present	0 (0%)
Not present	71 (100%)
Physical Examination (n, %)	
Normal	71 (100%)
Abnormal	0 (0%)
Body Mass Index (n, %)	
Underweight	0 (0%)
Normal	25 (35.2%)
Overweight	24 (33.8%)
Obese	22 (31.0%)
Virology	
HBV DNA PCR quantitative (IU/ML) (Mean± SD)	2190243.99± 10151769.09
HBeAg (n, %)	
Positive	8 (11.3%)
Negative	63 (88.7%)
Biochemistry	
ALT U/L (Mean± SD) [Normal range: 7 to 55 U/L]	49.66±98.16
AST U/L (Mean± SD) [Normal range: 8 to 48 U/L]	31.12±42.92
Serum Creatinine (mg/dL) (Mean± SD) [Normal range:0.84 to 1.21 mg/dL]	0.7014±0.277
FibroScan	
F0	6 (8.5%)
F1	13 (18.3%)
F2	5 (7.0%)
F3	6 (8.5%)
F4	9 (12.7%)
Ultrasound	
Normal	38 (53.5%)
Fatty	14 (19.7%)
Cirrhotic	11 (15.5%)

TAF feasibility

**Figure 1:**
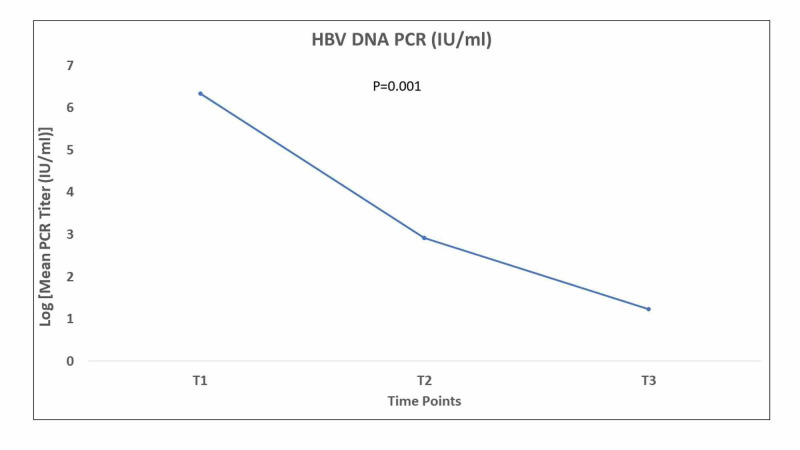
Mean HBV-DNA plasma level in log10 IU/mL at the three time points of the study HBV-DNA: hepatitis B virus deoxyribonucleic acid

The mean plasma HBV-DNA titer decreased from the time of the switch to six months later, from approximately 839 IU/mL to 17 IU/mL (p= 0.043) (Figure 1).

**Figure 2:**
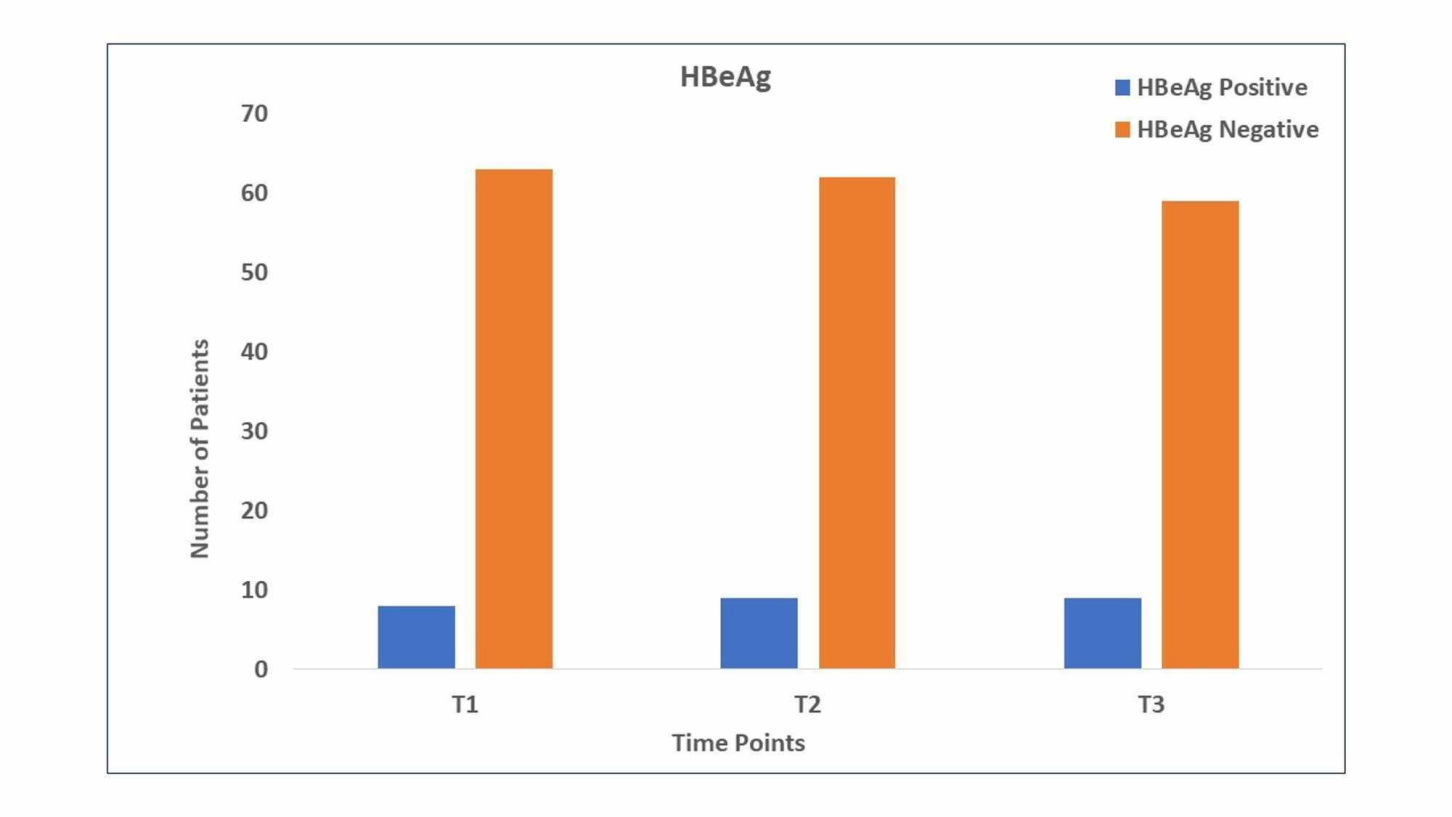
Numbers of HBeAg-positive and HBeAg-negative patients at the three time points of the study HBeAg: hepatitis B envelope antigen

The proportion of patients with positive HBeAg remained similar (12.7%, p>0.05) at six months after switching to TAF (Figure 2).

**Figure 3:**
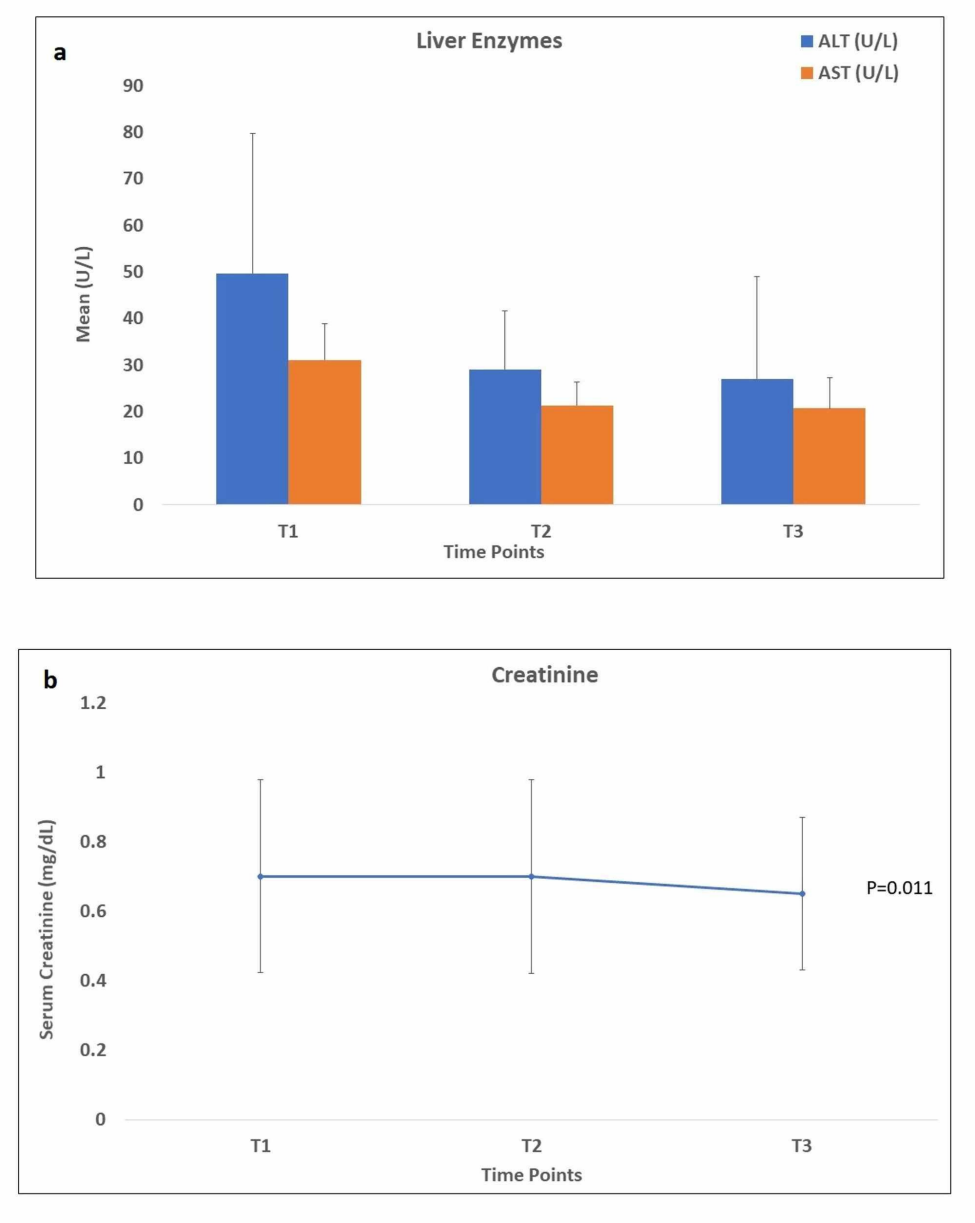
a) Mean levels of AST and ALT at the three time points of the study; b) Mean level of creatinine at the three time points of the study ALT: alanine transaminase; AST: aspartate transaminase

ALT and AST levels decreased slightly from the time of switching to TAF to six months later, but the changes were not significant (p= 0.328 and p= 0.410, respectively) (Figure 3a). Similar changes were observed for serum creatinine levels (p=0.472) (Figure 3b).

On the other hand, FibroScan results and ultrasound showed different percentages for their corresponding categories and significant difference (p=0.001) between the baseline (time before switching) and the endpoint. When comparing FibroScan results and ultrasound variables between the switching point and the endpoint, they showed a non-significant difference (p=0.485 and p=0.406), respectively (Table 2).

**Table 2:** Fibrosis score and ultrasound variables of HBV patients measured at three points in time T1- 1st time point (At the baseline, on the last medication administered just before switching to TAF) T2- 2nd time point (at the time of switch) T3- 3rd time point (6± 0.5 months after switching to TAF) * For the comparison between the first and the third point in time (McNemar testing using binomial distribution) ** For the comparison between the second and the third point in time (McNemar testing using binomial distribution) ^_a_^_One patient was excluded from this point due to the missing data_ _HBV: hepatitis B virus_

	T1 (n=71)	T2 (n=71)	T3 (n=70)^a^	P-value*	P-value**
Fibrosis Score (n, %)					
F0	6(8.5%)	3(4.2%)	4(4.6%)	0.001	0.485
F1	13(18.3%)	11(15.5%)	8(11.3%)		
F2	5(7.0%)	5(7.0%)	3(4.2%)		
F3	6(8.5%)	3(4.2%)	3(4.2%)		
F4	9(12.7%)	6(8.5%)	5(7.0%)		
Ultrasound (n, %)					
Normal	38(53.5%)	32(45.1%)	33(46.5%)	0.001	0.406
Fatty	14(19.7%)	13(18.3%)	15(21.1%)		
Cirrhotic	11(15.5%)	11(15.5%)	11(15.5%)		

**Figure 4:**
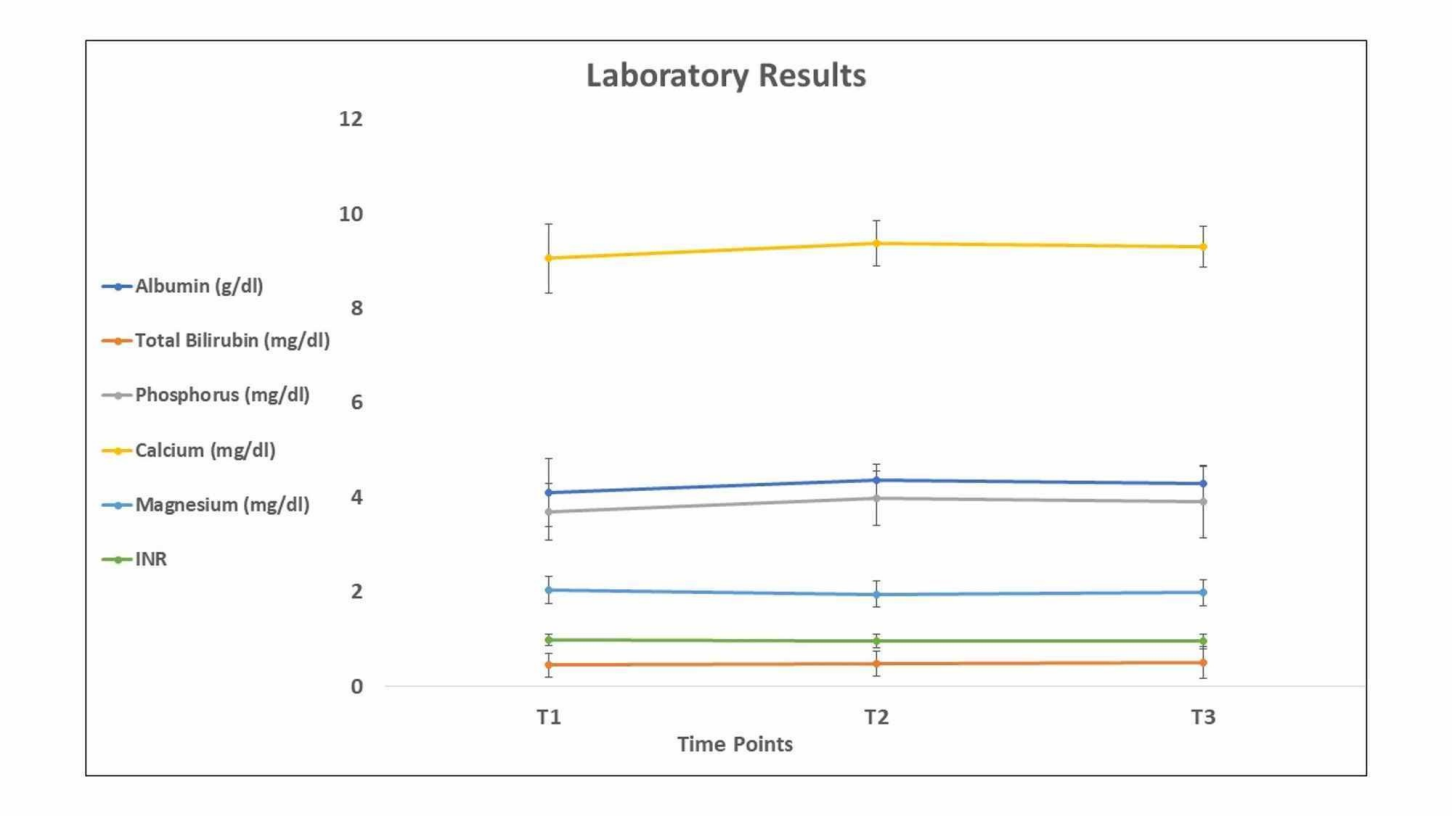
Biochemical parameters tested at the three time points of the study

The changes of several biochemical parameters tested at different time points are presented in Figure 4

**Figure 5:**
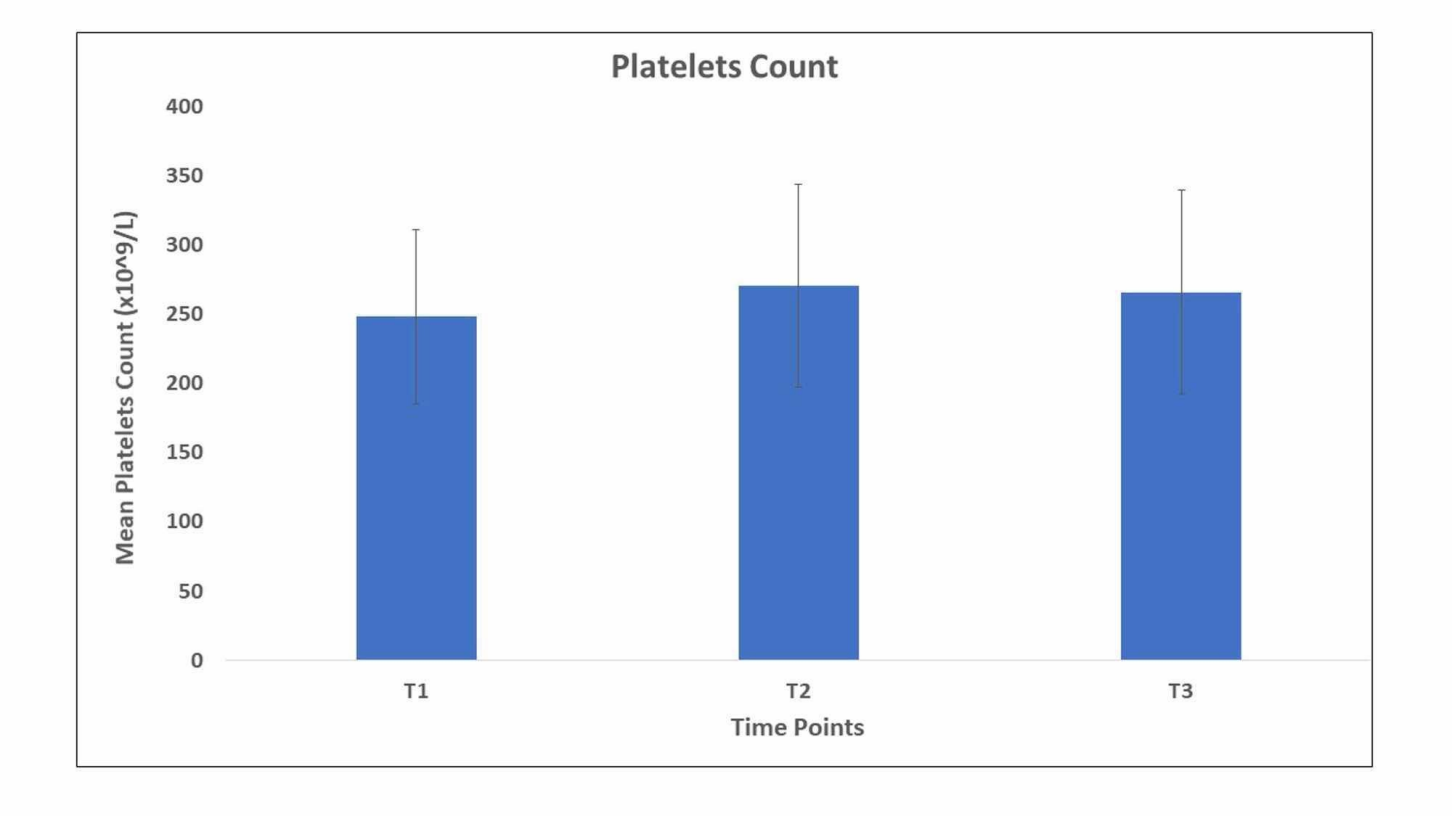
Mean platelets count at the three time points of the study.

Mean albumin levels remained within the normal range (3.4-5.4g/dl); however, there was a non-significant increase (p=0.05) in the mean level from 4.11 at the baseline (T1) to 4.30 at the third point in time (T3). There was a significant difference between T1 and T3 in terms of phosphorus and calcium serum levels (p = 0.02 and p = 0.013, respectively). Regarding the magnesium, INR, and total bilirubin levels, there was no significant difference among the time points. The same was observed for the number of platelets, which was consistently within the normal range; however, there was a significant increase between the first and third points in time (p = 0.004) (Figure 5).

Reasons for switching to TAF - adverse events

The top reason for switching to TAF was the TDF unavailability (81.7%), followed by side effects from other HBV antiviral agents (14.1%). Other reasons included the lack of efficacy of antiviral agents (1.4%), safety concerns (1.4%), and physician preference (1.4%). There was no report of any adverse events due to medication across all study periods.

Co-morbidities and concomitant medications

Diabetes mellitus was present in 16.9% of the enrolled population, followed by hypertension in 9.9%, hyperlipidemia and hypothyroidism in 4.2%, and epilepsy in 1.4% of the patients. All co-morbidities were present at baseline (T1). Patients enrolled in the study were on multiple medications. The most frequently reported indications for concomitant medications were agents for diabetes mellitus management (21.1%), followed by agents for hypertension (11.3%) and benign prostatic hyperplasia (8.5%). Other diseases requiring medications included hyperlipidemia, hypothyroidism, irritable bowel syndrome, urinary incontinence, and depression. The top medications taken by the patients were metformin (n=10), insulin (n=4), tamsulosin (n=4), amlodipine (n=3), and esomeprazole (n=3). Table 3 shows the medications taken by the patients with their corresponding dose, frequency, and route of administration.

**Table 3:** Concomitant medication throughout the study period

Indication	Medication Name	Patients, n	Dose	Frequency	Route	Status
Diabetes Mellitus	Metformin	10	500 mg	Twice daily	Oral	Ongoing
	Insulin	4	Variable	Twice daily	SC	Ongoing
	Sitagliptin	1	100 mg	Once daily	Oral	Ongoing
Hypothyroidism	Levothyroxine	3	Variable	Once daily	Oral	Ongoing
Hyperlipidemia	Rosuvastatin	2	10 mg	Once daily	Oral	Ongoing
	Atorvastatin	1	10 mg	Once daily	Oral	Ongoing
Irritable Bowel Syndrome	Mebeverine	1	200 mg	Twice daily	Oral	Ongoing
Hypertension	Telmisartan	1	40 mg	Once daily	Oral	Ongoing
	Indapamide	1	1.5 mg	Once daily	Oral	Ongoing
	Amlodipine	3	5 mg	Twice daily	Oral	Ongoing
	Metoprolol	1	25 mg	Twice daily	Oral	Ongoing
	Captopril	1	37.5 mg	Three times	Oral	Ongoing
	Perindopril	1	2.5 mg	Once daily	Oral	Ongoing
Epilepsy	Valproic acid	1	750 mg	Once daily	Oral	Ongoing
Peptic Ulcer Disease	Esomeprazole	3	20 mg	Twice daily	Oral	Ongoing
Benign Prostatic Hyperplasia	Tamsulosin	4	4 mg	Once daily	Oral	Ongoing
	Finasteride	2	5 mg	Once daily	Oral	Ongoing
Urinary Incontinence	Tolterodine	1	2 mg	Twice daily	Oral	Ongoing
Depression	Escitalopram	1	20 mg	Twice daily	Oral	Ongoing

.

## Discussion

TAF targets OATP1B3 and ATP1B1 transporters, using them through passive diffusion, to enter the primary hepatocytes, where it is converted to TFV by carboxylesterase 1 [19]. The active metabolite of TFV, TFV-DP, which results from the phosphorylation of the intracellular TFV, inhibits the replication of HBV DNA through reverse transcriptase [20]. The stability of TAF and its ability to penetrate target cells reduces circulating TFV levels [21]. Consequently, the administration of lower effective TAF doses is allowed in comparison to its predecessor, TDF [22]. Moreover, TDF can be hydrolyzed in the gut and plasma, but TAF is not affected. In terms of drug interaction, no major drug interactions were observed in the case of TAF while in TDF, regimens containing ritonavir-boosted protease inhibitors were observed to increase the circulating levels of TDF and TDF-associated nephrotoxicity [22].

In this real-world observational study, we showed that six months of TAF therapy maintained the clinical stability of HBV-infected patients. The administration of TAF reduced HBV DNA plasma levels significantly (p = 0.043); however, it maintained ALT and AST levels within the normal range six months after switching to TAF without any significant change or increase in their levels. Moreover, the frequencies of FibroScan domains (F0-F4) were decreased significantly (p=0.001). Similarly, TAF maintained stable kidney function throughout the study duration, which was reflected by serum creatinine levels. These findings indicate that TAF can be a suitable substitute for the current antiviral regimens, including TDF in patients with HBV infection without loss of efficacy. Moreover, these higher ALT normalization rates can be translated into greater histological improvement with less inflammatory activity and reduced fibrosis progression, or a reduced risk of long-term complications such as HCC [23]. Our findings are in agreement with the results of the two-phase III multicenter randomized controlled trials comparing TAF and TDF reported by Buti et al. and Chan et al. [24-25]. Both studies showed that patients treated with TAF as compared to TDF achieved similar inhibition of HBV replication, which was maintained after switching patients from TDF to TAF while TAF offered higher ALT normalization rates before and after switching. There was no significant difference between the two drugs in terms of HBeAg loss or HBeAg seroconversion. However, another single-center study conducted on 48 chronic HBV patients compared patients who switched from entecavir to TAF and patients who continued on entecavir. This study reported that changes in the serum ALT levels and serum HBsAg levels were comparable between the two patient groups [26].

TAF, as compared to TDF, was shown to have favorable changes in serum creatinine levels and estimated glomerular filtration rate (eGFR) as well as in hip and spine bone marrow density (BMD) over the first two years of therapy (p=0.001). Interestingly, both eGFR and BMD improved when patients were switched from TDF to TAF in those phase III trials [24-25]. BMD was not monitored in our study while we did not observe a significant improvement in serum creatinine levels after switching patients to TAF. In a study conducted on chronic HBV patients, Hagiwara et al. found that switching to TAF after entecavir or continuing on entecavir did not impact the renal function (estimated glomerular filtration rate (eGFR) levels) of both patient groups [26]. Our findings could be related to the different HBV agents instead of TDF only used by the patients before switching to TAF, the small number of patients included in our study increasing the possibility of a type II error and the relatively short duration of six months of TAF therapy. The European Association for the Study of the Liver (EASL) recommends TAF, instead of TDF, in patients with three specific risk factors: age >60 years, risk of bone disease, and renal problem [27]. As antiviral therapy is intended to last for a long time in the majority of chronic HBV patients who do not achieve HBsAg loss, it is reasonable to recommend to preferentially select drugs less prone to induce adverse bone and renal effects [28]. Therefore, all the above patients could benefit from the switch from TDF to TAF.

The availability of TAF and the side effects of the other HBV drugs seem to be the leading causes of switching chronic HBV patients to TAF in KSA. Blier et al. reported that switching medications during the management of the patient is a common practice; this switching may be due to the cost of previous medication or the availability of new medication with approved efficacy and improved safety [29]. Regarding safety, TAF showed a promising profile with no recorded drug-related adverse events during six months of therapy, although it was used in patients with several co-morbidities who were using many concomitant medications. In terms of pregnancy, TAF is classified as FDA pregnancy category B drugs, as no human data about the use of this drug in pregnancy is available [23]. All co-morbidities were already present at the onset of the last HBV therapy before TAF and none developed during the administration of TAF. In contrast, other studies reported some adverse drug reactions (ADRs) such as nausea, back pain, fatigue, and headache [24-25], which, however, were not related to the study drug. None of our patients discontinued TAF, whereas only 1% of patients discontinued treatment with TAF as compared to 1.2% in the TDF group in the phase III TAF registrational trials [24-25]. All these data indicate that TAF is a well-tolerated therapeutic agent [24].

Our study has some limitations: (1) a selection bias can be identified, as all patients were selected from one center and there was no representation of other races in Saudi Arabia; (2) the relatively small sample size of the study population, which can be explained by our restricting criteria and conducting the study in a single-center; (3) the high rate of missing records, including adverse events, which hindered drawing useful conclusions in some of the variables; (4) although patients with poor renal function would have been useful to include in this study; however, we could not find enough data to be considered.

## Conclusions

Our findings suggest that TAF is a feasible, well-tolerated, and safe alternative in chronic HBV patients who are treated with TDF, ETV, LAM, and adefovir. TAF maintained significant viral suppression and higher AST and ALT normalization rates. The advantage of TAF in the reduction of serum HBsAg levels should be further evaluated, especially in patients with HBV infection without underlying cirrhosis.
